# EndoNet: A Multiscale Deep Learning Framework for Multiple Gastrointestinal Disease Classification via Endoscopic Images

**DOI:** 10.3390/diagnostics15162009

**Published:** 2025-08-11

**Authors:** Omneya Attallah, Muhammet Fatih Aslan, Kadir Sabanci

**Affiliations:** 1 Department of Electronics and Communications Engineering, College of Engineering and Technology, Arab Academy for Science, Technology and Maritime Transport, Alexandria 21937, Egypt; 2 Wearables, Biosensing, and Biosignal Processing Laboratory, Arab Academy for Science, Technology and Maritime Transport, Alexandria 21937, Egypt; 3 Department of Electrical and Electronics Engineering, Karamanoglu Mehmetbey University, Karaman 70100, Türkiye; mfatihaslan@kmu.edu.tr (M.F.A.); kadirsabanci@kmu.edu.tr (K.S.)

**Keywords:** convolutional neural networks, feature fusion, gastrointestinal disease classification, minimum redundancy maximum relevance, non-negative matrix factorization

## Abstract

**Background:** Gastrointestinal (GI) disorders present significant healthcare challenges, requiring rapid, accurate, and effective diagnostic methods to improve treatment outcomes and prevent complications. Wireless capsule endoscopy (WCE) is an effective tool for diagnosing GI abnormalities; however, precisely identifying diverse lesions with similar visual patterns remains difficult. **Methods:** Many existing computer-aided diagnostic (CAD) systems rely on manually crafted features or single deep learning (DL) models, which often fail to capture the complex and varied characteristics of GI diseases. In this study, we proposed “EndoNet,” a multi-stage hybrid DL framework for eight-class GI disease classification using WCE images. Features were extracted from two different layers of three pre-trained convolutional neural networks (CNNs) (Inception, Xception, ResNet101), with both inter-layer and inter-model feature fusion performed. Dimensionality reduction was achieved using Non-Negative Matrix Factorization (NNMF), followed by selection of the most informative features via the Minimum Redundancy Maximum Relevance (mRMR) method. **Results:** Two datasets were used to evaluate the performance of EndoNer, including Kvasir v2 and HyperKvasir. Classification using seven different Machine Learning algorithms achieved a maximum accuracy of 97.8% and 98.4% for Kvasir v2 and HyperKvasir datasets, respectively. **Conclusions:** By integrating transfer learning with feature engineering, dimensionality reduction, and feature selection, EndoNet provides high accuracy, flexibility, and interpretability. This framework offers a powerful and generalizable artificial intelligence solution suitable for clinical decision support systems.

## 1. Introduction

Gastrointestinal (GI) diseases are a group of diseases that include many different clinical conditions, such as peptic ulcers, polyps, reflux, erythema, and inflammation, and constitute a serious health burden worldwide. Early diagnosis of GI diseases is critical for the success of treatment and improvement of quality of life [1]. According to global health reports, GI diseases affect millions of individuals per year and cause high mortality and morbidity rates, especially in developing countries [2]. Early diagnosis of these diseases is of extremely critical importance not only for increasing the treatment success but also for reducing the risks of complications, improving the quality of life, and reducing the healthcare costs. Endoscopy is widely used as the gold standard method in the diagnosis of GI diseases and allows detailed analysis of the stomach and intestinal walls based on direct visual examination. However, the fact that this process is manual and operator-dependent due to its nature leads to various limitations on diagnostic accuracy. Subjective factors such as attention, experience level, and interpretation of the physician during the endoscopic evaluation may cause the same lesion to be evaluated in different ways by different clinicians [3,4]. In addition, factors such as the increasing number of patients, specialist shortage, and time pressure make manual endoscopic evaluations more difficult and increase the need for decision support systems [5].

In recent years, deep learning (DL) techniques, especially convolutional neural networks (CNNs), have achieved groundbreaking achievements in the field of medical image processing [6]. CNN-based systems offer much more powerful and generalizable models compared with classical image processing approaches, thanks to their ability to automatically learn higher-level semantic structures starting from low-level edge-texture information in images [7]. In addition, CNN models have also attracted attention for their ability to learn complex structures in endoscopic images [8,9]. However, the characteristics obtained with a single CNN architecture may not always be sufficient to represent all clinical variations because different CNN architectures tend to learn features at different scales and depths. Therefore, combining information from multiple CNN structures stands out as a powerful strategy for improving diagnostic accuracy [10]. Furthermore, conventional methods for GI classification using DL models typically gather attributes from an individual CNN layer, failing to fully exploit the intricate representations of attributes available in multi-layered CNN frameworks. This limitation restricts analytical detail and may overlook crucial information that might boost diagnostic outcomes [11].

Deep feature representations obtained from a large number of CNNs are often high-dimensional and, when given directly to classification algorithms, both increase the computational cost and bring with them the risk of overfitting [12]. In order to overcome this problem, various size reduction and feature selection techniques have been developed. Dimensionality reduction and attribute selection techniques not only increase the model’s efficiency, they also directly affect the classification success. In this context, the Non-Negative Matrix Factorization (NNMF) method produces more meaningful and interpretable representations, especially in medical images. By learning part-based representations of the data, it offers more interpretable and low-dimensional representations [13]. On the other hand, the Minimum Redundancy Maximum Relevance (mRMR) method selects information-rich but independent attributes in systems that contain a large number of features, in particular. Thus, the generalizability and classification success of the model are improved [14,15].

In this study, EndoNet, a multi-stage framework that includes multiple CNN architectures, multi-layer feature extraction, multi-stage feature fusion, size reduction, and attribute selection steps, is proposed. The proposed method was evaluated on a multiple GI disease dataset consisting of eight classes called Kvasir v2 [16,17]. First, feature extraction was performed from two different deep layers (Layer I, Layer II) from pre-trained CNNs such as Inception, Xception, and ResNet101. Layer I deep features were reduced by NNMF, then features were decoupled between layers, and then networks were applied in two stages. Recent research shows that information theory-based attribute selection techniques, such as mRMR, increase the classification accuracy in DL-based systems and make the model simpler [18,19]; therefore, attribute selection was made using mRMR. Finally, the diagnosis was made with seven different machine learning (ML) classifiers.

The unique contribution of this study is to present a multi-stage architecture that maximizes classification success by systematically combining multi-layer features extracted from DL models at both inter-layer and inter-network levels. The representations obtained from different layers of each CNN model (Inception, Xception, ResNet101) were first evaluated separately. Then, these representations were fused both at the layer level and at the network level. This structure made it possible to learn a richer and more detailed representation by combining the complementary features of different architectures and different layers, rather than the limited perspective of a single deep model and only one deep layer. These combined high-dimensional features were reduced using NNMF, and then the most informative and independent features were selected with mRMR to reduce the risk of over-fitting the model. Then, classification operations were performed using seven different traditional ML algorithms. The proposed EndoNet framework goes beyond just the classic transfer learning steps and offers a comprehensive approach in which feature engineering, size reduction, and selection strategies are integrated with deep features. In this aspect, this study reveals a remarkable DL-based diagnostic support system both in terms of model performance and clinical applicability. The findings obtained provide a powerful, flexible, and interpretable AI solution that can be used especially within the scope of clinical decision support systems that require high accuracy and generalizability.

The article is organized as follows: In the second section, previous studies are compiled, and a literature review is presented. In the third section, the proposed DL-based diagnostic framework named EndoNet is introduced step by step. In the fourth section, the experimental setup is described, and the classification results are explained. In the fifth section, the obtained results are discussed in the light of previous studies. In the sixth section, the general conclusions of the study and its potential contributions to clinical decision-support systems are summarized.

## 2. Literature Review

The latest developments in DL, particularly with CNNs, have transformed the domain of medical imaging. CNNs have emerged as the predominant method for the analysis of medical images [20], proficiently addressing a range of illnesses such as lung and colon cancers [21,22], cervical cancer [23], and skin cancer [24,25]. This achievement has inspired researchers to employ CNNs for the classification of various GI diseases.

In the past two years, ensemble and multimodal approaches in GI CAD have gained significant attention. For instance, Siddiqui, et al. [26] proposed CG-Net, a custom-designed CNN architecture aimed at the multi-class classification of GI diseases using endoscopic images. Unlike typical transfer learning approaches, CG-Net is built from scratch and consists of 24 layers, including multiple convolutional, ReLU, batch normalization, max-pooling, and dropout layers, specifically structured to optimize learning for GI image features. The model was trained and tested on the Kvasir v2 dataset, which includes 8000 images across eight disease categories. CG-Net achieved a remarkably high classification accuracy of 99.30%, surpassing state-of-the-art CNN architectures such as ResNet50, DenseNet201, MobileNetV2, and GoogleNet under identical experimental conditions. The study emphasizes architectural simplicity, improved generalization without overfitting, and suitability for real-time diagnostic applications without relying on pretrained models. However, this study does not employ multi-model or multi-layer feature fusion, nor does it utilize any structured feature selection strategy, which are key components of our proposed framework.

Siddiqui, et al. [27] proposed a deep ensemble model combining NasNet-Mobile and EfficientNet for the classification of gastrointestinal diseases using endoscopic images. The model leverages transfer learning to address challenges such as limited annotated data and imbalanced datasets. It was trained on the HyperKvasir dataset and tested on the v1 and v2 datasets, which include images across eight disease categories. The ensemble model achieved high classification accuracies of 98.45% and 97.83% on the test datasets, outperforming individual state-of-the-art models. The study highlights the effectiveness of weighted voting ensemble techniques, computational efficiency, and improved generalization for real-world medical applications. However, this study focuses on model-level ensembling and does not explore hierarchical feature-level fusion or multi-layer representation integration as proposed in our approach.

In another recent study, Werner, et al. [28] proposed a hardware-efficient ensemble model combining unsupervised, semi-supervised, and supervised anomaly detection techniques using MobileNetV3 as the backbone architecture. The model integrates an autoencoder for unsupervised reconstruction, a classification head for semi-supervised learning, and a standard image classifier, with predictions combined via a Random Forest or SVM. Evaluated on the Kvasir-Capsule and Galar datasets, the largest publicly available WCE datasets, the ensemble achieved Area Under the Curve (AUC) scores of 76.86% and 76.98%, respectively, while significantly reducing parameter counts (to ~4 million) compared with state-of-the-art models like ResNet152 and ViT. The study highlights the model’s suitability for low-power edge devices, prioritizing anomaly detection sensitivity to enable real-time, energy-efficient analysis in capsule endoscopy systems. However, the focus of this study is anomaly detection in capsule endoscopy, and it does not incorporate supervised multi-layer feature fusion or deep feature selection mechanisms as applied in our CAD-based classification framework.

Dermyer, et al. [29] introduced EndoDINO, a foundation model pre-trained on a large and diverse dataset of GI endoscopy images derived from 130,037 videos, totaling over 3.5 billion frames, unlike traditional methods that rely on natural image datasets like ImageNet or smaller endoscopic datasets, EndoDINO leverages self-supervised learning (DINOv2 methodology) to train ViT models with up to 1 billion parameters. The model was evaluated on tasks such as anatomical landmark classification, polyp segmentation, and Mayo endoscopic scoring (MES) for ulcerative colitis, achieving state-of-the-art performance even with simple decoder heads. For example, in polyp segmentation, EndoDINO ViT-L/14 achieved a mDice score of 0.909, outperforming prior models, while in 4-class MES, the ViT-g/14 variant attained an AUROC of 0.942. The study highlights the advantages of domain-specific pre-training, including improved generalizability, reduced need for large labeled datasets, and the ability to support multiple downstream tasks efficiently, making it suitable for real-time clinical applications. Key innovations include advanced data curation techniques (e.g., deduplication and hierarchical clustering) and the scalability of the model to diverse GI endoscopy tasks without task-specific fine-tuning. However, this study focuses on large-scale foundation model training and does not address feature-level fusion or classical ML-based classification, which are central to our lightweight and interpretable CAD framework.

Sivari, et al. [30] proposed a hybrid stacking ensemble model based on DL for the detection and classification of GI system findings from endoscopic images. In the study, five-fold cross-validation was applied with three new CNN models, and the estimates of these mode-hands were transferred to an ML classifier at the second level. This two-level approach provided significant performance improvement compared to the individual DL models, with over 98% accuracy and Matthews correlation coefficient (MCC) in both the KvasirV2 and HyperKvasir datasets. In addition, it has been shown that the results obtained by the McNemar statistical test are statistically significant. However, unlike our method, this study does not perform hierarchical deep feature fusion or apply multi-stage feature selection techniques to optimize the final classification performance.

Gunasekaran, et al. [31] aimed to achieve high accuracy in the classification of GI diseases on endoscopic images by developing a DL-based ensemble model called GIT-Net. In the study, three pre-trained models (DenseNet201, InceptionV3, and Res-Net50) were used to classify eight different GI diseases in the Kvasir v2 dataset. The individual accuracy of these models was reported as 94.54%, 88.38% and 90.58%, respectively. GIT-Net has realized the final classification by combining the estimates of these three models with the model average and weighted average methods. While 92.96% accuracy was obtained by the model average method, the weighted average ensemble model showed the best performance with 95.00% accuracy. The results show that the ensemble approach is able to classify more accurately the samples that the individual models misclassify. However, this model focuses solely on output-level ensembling without utilizing intermediate deep features, multi-layer fusion, or hybrid ML-based classification as performed in our framework.

Lumini, et al. [32] proposed a new method to improve the performance of deep ensemble models in the field of semantic segmentation by randomly modifying the activation functions inside the model. In the study, the performance of the communities created by randomly selecting activation functions in each network was evaluated using the DeepLab architecture and different backbone networks. A significant level of success was attained using the suggested method in the trials that were carried out using the Kvasir-SEG dataset. These results included a dice coefficient of 0.888 and an average Intersection over Union (mIoU) value of 0.825. In addition, in different segmentation tasks such as skin detection, this ensemble approach has left the best available methods behind. The results show that randomization of activation functions increases the diversity and performance of ensemble models. This approach makes an important contribution to improving the accuracy and reliability of DL-based segmentation, especially in sensitive applications such as medical image analysis. However, unlike our study, this work focused on segmentation tasks and activation-level randomization, without exploring multi-layer feature fusion or traditional ML classifiers for enhanced interpretability in classification tasks.

Khan, et al. [33] developed an automated framework called GestroNet for the identification and classification of GI diseases. In this study, after improving the images with contrast enhancement, segmentation of infected regions was performed using deep saliency maps. The MobileNet-V2 model, which had been pre-trained and fine-tuned by transfer learning, was used to extract features following segmentation. The hyperparameters of the model were determined using Bayesian optimization, and then attribute extraction was performed using average pooling. The best ones among the obtained attributes were selected with the hybrid whale optimization algorithm, and classification was performed with the Extreme Learning Machine classifier at the last stage. GestroNet achieved 98.20%, 98.02%, and 99.61% accuracy rates in CUI Wah datasets, Kvasir v1, and Kvasir v2, respectively, and showed higher success compared with existing methods. However, this study applies feature selection after a single-model feature extraction and does not incorporate hierarchical multi-layer fusion from multiple deep networks, as proposed in our method.

Naz, et al. [34] proposed a hybrid method using XcepNet23 and ResNet18-based ensemble attributes for the diagnosis and classification of GI abnormalities. In the study, hybrid contrast stretching algorithms were applied for image enhancement, and then deep attributes were extracted from ResNet18 and the proposed XcepNet23 models with transfer learning. The obtained deep attributes were combined with texture attributes to create an ensemble attribute vector. This vector was reduced to the best subset of attributes by optimization algorithms such as Binary Dragonfly Algorithm (BDA), particle swarm optimization (PSO), and Moth–Flame Optimization (MFO). Classification was performed using the Q_SVM method, and the Hybrid dataset had 100% accuracy, while the Kvasir V1 dataset had 99.24% accuracy. The findings demonstrate that, in comparison to the existing approaches, the suggested approach offers greater accuracy. However, this study did not utilize multi-layer deep representations or comparative feature extraction from different levels of CNNs, which are key components of our proposed multi-level fusion strategy.

El-Ghany, et al. [35] developed a DL-based, computer-aided diagnostic system for the detection of GI diseases using wireless capsule endoscopy (WCE) images. In this study, the Intelligent Learning Rate Controller (ILRC) mechanism, which dynamically adjusts the learning rate during model training, was introduced, and thus, the prevention of excessive learning has been achieved with faster convergence of the model. Four different DL models— ResNet101v2, EfficientNet-B0, InceptionResnetV2, and InceptionV3—were subjected to ILRC. These models were further developed with transfer learning, layer freezing, fine-tuning, modern editing, and residual-learning techniques. In tests conducted on Kvasir Capsule and Kvasir v2, the accuracy rate of the models used with ILRC reached 99.91% and 98.06%, respectively, and performed better than existing methods. This approach significantly improves clinical diagnostic processes by providing high accuracy and efficiency in the automatic detection of GI diseases on WCE images. However, while this study focuses on dynamic training optimization, it does not explore structured multi-layer feature fusion or deep feature selection across multiple CNN architectures, which are central to our framework.

Tsai and Lee [36] combined the Gradient-weighted Class Activation Mapping (Grad-CAM) method with a dynamic weighted ensemble learning framework in order to increase accuracy and interpretability in the classification of GI diseases in endoscopic images. Using the Kvasir v2 dataset, the study fine-tuned three popular CNN models—DenseNet201, Inception V3, and VGG19—and produced a dynamic ensemble model with optimized weights of 0.4, 0.4, and 0.2. This ensemble model provided higher accuracy (0.91) compared with the singular models, especially in complex categories such as Normal-Z-Line and esophagitis. GradCAM visualizations have shown that the model focuses on clinically significant attributes and improves the interpretability of its decisions. While the dynamic ensemble approach improves classification success and explicability, it has been noted that further improvements are needed in subtle and ambiguous cases. This study reveals that dynamic collective learning shows promise in terms of both accuracy and clinical applicability in medical imaging. However, unlike our study, it did not perform structured feature extraction across multiple deep layers or integrate deep features with traditional ML classifiers to enhance both performance and interpretability.

In the study carried out by Janutėnas and Šešok [37], two innovative modules were added to the classical StarGAN architecture for endoscopic image enhancement: Perspective Transformation and Angle of View Attention modules. These improvements have enabled the highlighting of particularly diagnostically critical regions and the production of images from different angles. In the evaluations carried out on the Kvasir v2 dataset, the approach developed increased the classification accuracy by 0.7% and 0.63%, respectively, compared with basic models such as VGG-16 and EfficientNet-B7. The study showed that generative augmentation techniques can offer an important contribution to the medical imaging field with limited data. However, unlike our study, this work focused on image augmentation using GAN-based methods rather than on deep feature fusion and structured selection across multiple CNNs to enhance classification performance and model interpretability.

Khan, et al. [38] proposed a unique DL architecture for the classification and localization of GI diseases from WCE images. In the study, solutions to problems such as in-class similarity, unbalanced data distribution, and high computational costs faced by traditional deep networks were introduced. The architecture developed for this purpose has been created by combining Sparse Convolutional DenseNet201 with Self-Attention (SC-SAN) and CNN-GRU models at a network level. Entropy-controlled Marine Predators Algorithm (EMPA) was used for feature selection, and Bayesian optimization (BO) was used for hyperparameter optimization; the classification process was performed with Shallow Wide Neural Network (SWNN). In the experiments conducted on Kvasir-V1 and Kvasir-V2 datasets, 99.60% and 95.10% accuracy were obtained, respectively. The results obtained show that the proposed structure is superior to existing methods both in terms of classification performance and computational efficiency. However, this study focused on a custom hybrid architecture and metaheuristic optimization, whereas our approach emphasizes structured deep feature fusion across multiple pre-trained CNNs and multi-layer abstraction, improving both classification and interpretability through conventional ML integration.

Kamble, et al. [39] proposed an interpretable and high-accuracy DL model in the multi-class classification of GI endoscopic images. In the study, 8000 labeled endoscopic images taken from the Kvasir dataset were divided into eight different classes using an EfficientNetB3-based architecture, and 94.25% accuracy, 94.29% sensitivity, and 94.24% recall rate were obtained without the need for data augmentation methods. In order to explain the decision mechanism of the model, LIME (Local Interpretable Model-agnostic Explanation) saliency maps were used to visualize which image regions the model focuses on. This approach shows that DL models can offer both high performance and clinically meaningful interpretability in medical image analysis. However, unlike our framework, this study does not employ multi-model, multi-layer deep feature fusion or structured feature selection mechanisms, which limits the depth of learned representations and classifier integration flexibility.

Mohapatra, et al. [40] developed an intelligent healthcare system based on wavelet transform and deep CNN for the detection of GI diseases. In the study, eight different GI system image classes in the Kvasir v2 dataset were used, and the attributes were extracted using discrete wavelet transform after the images were pre-processed. The obtained attributes were transferred to CNN models for two-stage classification, and 97.25% accuracy was achieved in the first stage, and 93.75% accuracy was achieved in the second stage. The success of the model has also been confirmed by criteria such as sensitivity, recall, specificity, and F1 score. In addition, it was stated that the proposed method performs better compared with previously published methods in similar datasets. This study showed that DL-based approaches enriched with wavelet transform are effective in the automatic and sensitive detection of GI diseases. Similarly, Attallah et al. [41] employed discrete cosine transform and discrete wavelet transform with CNNs for classifying multiple GI diseases using the Kvasir v1 dataset, reaching an accuracy of 97.3%. However, both studies emphasize hand-crafted transform domain features in conjunction with CNNs, whereas our method focuses on hierarchical deep feature extraction from multiple CNN layers and models, followed by structured selection and ML-based classification to enhance both performance and interpretability.

Khan, et al. [42] proposed a deep CNN-based method for the accurate classification of GI system syndromes. In the study, deep features were extracted from endoscopic images with pre-trained networks such as Darknet-53 and Xception, and then attribute optimization was performed with the Binary Dragonfly Algorithm (BDA). The obtained attributes were combined and transferred to the Ensemble Subspace K-Nearest Neighbors (ESKNN) classifier. The model was tested on the Kvasir-v2 benchmark dataset and the COMSATS University Islamabad (CUI) Wah special dataset. A total of 98.25% accuracy was achieved in the Kvasir-v2 dataset, and 99.90% accuracy was achieved in the CUI Wah dataset. In addition, performance comparisons were made with different attribute selection techniques, such as genetic algorithm, particle swarm optimization, salp swarm algorithm, sine cosine algorithm, and gray wolf optimization. The results show that the proposed model shows superior performance compared with existing methods and holds promise for automatic GI classification. Although this study performs feature fusion and metaheuristic selection, it does not utilize hierarchical multi-layer features from each CNN nor apply sequential dimensionality reduction followed by statistical selection as performed in our method, which enhances both interpretability and classification performance.

Cogan, et al. [43] developed a DL-based framework called MAPGI for the accurate detection of anatomical signs and diseased tissues in the GI tract. In the study, automatic and modular pre-processing steps such as edge lifting, contrast enhancement, filtering, color mapping, and scaling were applied to the endoscopic images in the Kvasir dataset. Pixel values were normalized with gamma correction for each image. Then, current deep neural network architectures such as Inception-ResNet-v2, Inception-v4, and NASNet were trained, and 98.48%, 98.45%, and 97.35% accuracy were achieved, respectively, in the validation data. The Inception-v4 model also stood out with a sensitivity of 93.8%, a recall of 93.9%, a specificity of 99.1%, an F1 score of 93.8%, and a Matthews correlation coefficient (MCC) of 92.9%. These results show that the MAPGI framework is able to detect both anatomical signs and diseased tissues with high accuracy, even in small and diverse medical image datasets. However, unlike our framework, this study did not incorporate structured multi-layer feature extraction and dimensionality reduction techniques (such as NNMF and mRMR) across multiple CNNs, nor did it explore traditional ML classifiers for enhanced interpretability.

When the current studies conducted in this field are examined, it is seen that classification is usually made using only one DL architecture, and attribute reduction and selection methods are mostly neglected. Furthermore, deep features were extracted from a single deep layer of the DL architecture. In addition, in many studies, classification is performed directly through the output of deep networks, and the integration of deep features with traditional ML methods has not been sufficiently investigated. These limitations in the literature have caused both the classification success and the interpretability of the model to remain limited. Although some recent studies, such as CG-Net [26], ensemble models based on NasNet-Mobile and EfficientNet [27], or EndoDINO [29] have introduced novel architectures or large-scale pre-training strategies, they still rely on either single-model pipelines, limited layer usage, or do not perform structured feature selection. Therefore, holistic approaches are needed, such as integrating attributes obtained from multiple CNN architectures and several deep layers, using effective attribute reduction and selection techniques, and evaluating these attributes with traditional classifiers. In this context, this study aimed to provide a more comprehensive and high-performance solution by targeting the gaps in the literature.

While the components used in the proposed framework, such as CNN-based feature extraction, dimensionality reduction, and classical ML classifiers, are individually well established, their combined implementation in this study is novel in terms of both architecture and integration strategy. Specifically, the fusion of multiple CNN models across different hierarchical layers, followed by a two-stage feature optimization (NNMF and mRMR), and final evaluation with traditional classifiers, has not been previously reported in GI-based CAD applications. This integrated approach provides a balanced trade-off between model accuracy, interpretability, and computational efficiency, which are critical for real-time clinical applicability.

Compared with these recent studies, the key advantages of the proposed framework can be summarized as follows:

- Multi-model feature fusion was performed not only at the network level but also across different hierarchical layers (shallow and deep), enhancing feature diversity;
- A two-stage attribute optimization was applied (NNMF followed by mRMR), ensuring dimensionality reduction and relevance filtering before classification;
- The final decision-making step was handled using traditional ML classifiers, providing improved interpretability and adaptability;
- The system is designed to be lightweight, avoiding the computational burden of large pre-trained or transformer-based models, making it suitable for real-time diagnostic support.

## 3. Methodology

This section describes in detail the proposed DL-based diagnostic framework designed for the classification of GI diseases using endoscopic images. The method integrates several deep neural networks (CNNs), extracts features from multiple internal layers, applies dimensionality reduction, and uses traditional ML classifiers to finalize the diagnosis. This study is a retrospective/prospective analysis aiming to develop and validate a DL-based diagnostic framework for classifying gastrointestinal diseases from endoscopic images. The primary outcome is the classification accuracy of the model across eight disease categories. Secondary outcomes include sensitivity, specificity, and interpretability measures.

While these processes may seem complex to non-technical readers, the workflow can be understood in a simplified way. First, multiple neural networks are employed to ‘analyze’ the visual characteristics in the endoscopic images, learning to recognize meaningful patterns that may indicate specific GI diseases. From the vast amount of information extracted, only the most important features are selected; those that provide the clearest distinction between disease types. These selected features are then used to make predictions about which disease class an image belongs to. This layered and filtered approach helps improve diagnostic accuracy while also reducing the risk of overfitting. Overall, the model simulates how a medical expert might focus on critical visual cues while disregarding irrelevant details. The aim of including this brief explanation is to enhance clarity and accessibility for readers with a clinical background who may not be deeply familiar with the technical aspects of artificial intelligence and ML.

### 3.1. Feature Reduction and Selection Approaches

#### 3.1.1. Non-Negative Matrix Factorization

NNMF is an effective statistical technique employed to decompose a non-negative matrix *V* into two smaller non-negative matrices, conventionally denoted as *W* and *H*, such that their product closely approximates the original matrix (*V ≈ WH*) [13]. This method is particularly efficacious for minimizing high-dimensional data by pinpointing essential features and diminishing complexity via interpretable, part-based representations of the dataset [44]. Unlike other decomposition methods, such as Principal Component Analysis (PCA), NNMF mandates that all elements in the resultant matrices be non-negative or zero due to its non-negativity criterion [45].

From a mathematical perspective, NNMF operates by utilizing an input matrix *V ∈ ℝ^(m × n)* and aiming to identify two non-negative matrices *W ∈ ℝ^(m × k)* and *H ∈ ℝ^(k × n)* that most accurately approximate *V* through their product. The objective is to reduce the divergence between the original and reconstructed matrices, typically quantified using the Frobenius norm:

(1) $$minimize\;\left\|{\left(V\;-\;WH\right)}^{2}\right\|\;,\;\left(\;W,\;H\;\geq \;0\right)\;$$

The Frobenius norm measures the total error of the approximation, while the factor *k*, typically significantly smaller than both *m* and *n*, facilitates a compact, lower-dimensional representation of the data [46].

Matrix W: An *m × r* matrix functioning as a basis vector, with each column denoting a distinct feature or component. 

Matrix H: An *r × n* matrix comprising coefficients that represent the combination of basis vectors from *W* to reconstruct the original data points. 

Enforcing non-negativity provides numerous practical advantages. A significant advantage is improved interpretability: the decomposition, being solely additive, corresponds more intuitively with human perception of parts constituting a whole. This constraint frequently yields sparse strategies, which can more efficiently elucidate significant patterns and structures within the data than traditional methods. These sparse representations enhance model generalization and reveal underlying relationships that may be challenging to identify in the complete, high-dimensional space.

#### 3.1.2. Minimum Redundancy Maximum Relevance

The mRMR strategy is used to perform an advanced feature selection (FS) process, with mutual information (MI) acting as the primary evaluation criterion [14]. This technique systematically filters feature subsets by pinpointing those that provide significant predictive capability for class labels while ensuring minimal redundancy among selected features. By doing so, mRMR enhances the selection process by prioritizing informativeness and uniqueness among features, thereby streamlining training, mitigating the risk of overfitting [47], and improving model efficiency and predictive accuracy.

MI measures the degree of interdependence across two variables, essentially indicating how much knowledge of one variable reduces uncertainty regarding the other. This is mathematically expressed by Equation (5), which includes both the marginal distributions P(A) and P(B), as well as the joint distribution P(A, B), as follows:

(2) $$I\left(A;B\right)=\sum_{b\in B}\sum_{a\in A}p\left(a,b\right)\log\left(\frac{p\left(a,b\right)}{p\left(a\right)p\left(b\right)}\right)\;$$

The maximum relevance measure, as delineated in Equation (6), is utilized to identify the most informative attributes concerning the target class. This criterion identifies features $X_{i}$ exhibiting the greatest mutual information with the target variable C. Relying exclusively on this principle may result in excessive redundancy, as highly relevant features may be similar or interrelated, thereby diminishing their overall effect on model performance. To resolve this, the minimum redundancy principle is implemented, as demonstrated in Equation (7), which assesses the pairwise mutual information $I\left(X_{i},\;X_{j}\right)$ between features, guaranteeing that chosen attributes yield complementary information [15].

(3) $$\max D\left(X,C\right);D=\frac{1}{\left|X\right|}\sum_{X_{i}\in X}I\left(X_{i},\;C\right)\;$$

(4) $$\min R\left(X\right);R=\frac{1}{\left|X\right|}\sum_{X_{i},\;X_{j}\in X}I\left(X_{i},\;X_{j}\right)\;$$

The integration of relevance and redundancy criteria constitutes the basis of the mRMR framework. Equation (8) embodies this dual optimization strategy and utilizes a greedy search algorithm to progressively assemble the optimal feature subset. In each iteration, the algorithm selects the feature from the remaining pool that maximizes the disparity between its relevance to the class label and its average redundancy with the features already included in set *S*. This balance guarantees a selection process that preserves high informativeness while eliminating redundancy, yielding a streamlined yet potent feature set that improves model generalizability and computational efficiency.

(5) $${max}_{X_{i}\notin S}\left[I\left(X_{i};C\right)-\frac{1}{\left|S\right|}\sum_{X_{j}\in S}I(X_{i},X_{j})\right]\;$$

### 3.2. Dataset Description

In order to develop and evaluate the proposed EndoNet framework in this study, two open-access endoscopic image datasets were used. The first dataset is named Kvasir v2, and the second dataset is called HyperKvasir. This section will present these two datasets.

#### 3.2.1. Kvasir v2 Dataset

**Figure 1:**
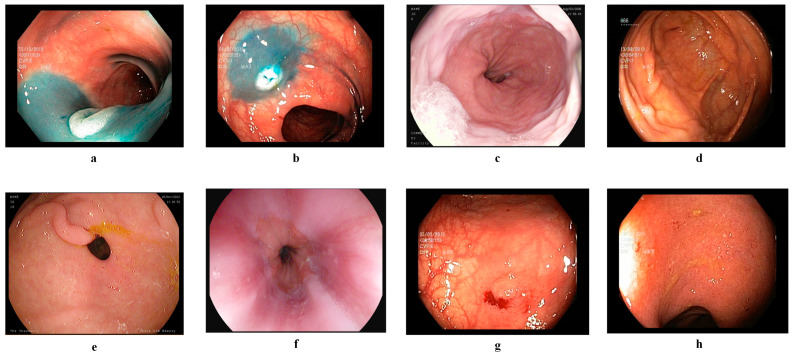
Examples of the endoscopic images of the Kvasir version 2 dataset: (**a**) dyed-lifted-polyps, (**b**) dyed-resection-margins, (**c**) esophagitis, (**d**) normal-cecum, (**e**) normal-pylorus, (**f**) normal-z-line, (**g**) polyps, and (**h**) ulcerative-colitis.

The Kvasir v2 dataset was created by the Simula Research Laboratory in Norway and made available to researchers via the Kaggle platform [17]. The dataset images represent various GI regions and have been labeled and reviewed by clinical experts for high diagnostic accuracy. The Kvasir v2 dataset contains a total of 8000 labeled color endoscopic images covering eight different GI disease classes. The resolutions of the images range from 720 × 576 to 1920 × 1072 pixels. Each class consists of 1000 samples, and the classes are dyed lifted polyps, dyed resection margins, esophagitis, normal cecum, normal pylorus, normal z-line, polyps, and ulcerative colitis. The images were obtained using standard white light endoscopy systems, labeled by specialized doctors, and their clinical validity was verified. Sample endoscopic images of the dataset are presented in Figure 1. With its features, such as balanced class distribution, high resolution, and expert labeling, the Kvasir v2 dataset stands out as a powerful benchmark source for multi-class endoscopic image classification.

#### 3.2.2. HyperKvasir Dataset

**Figure 2:**
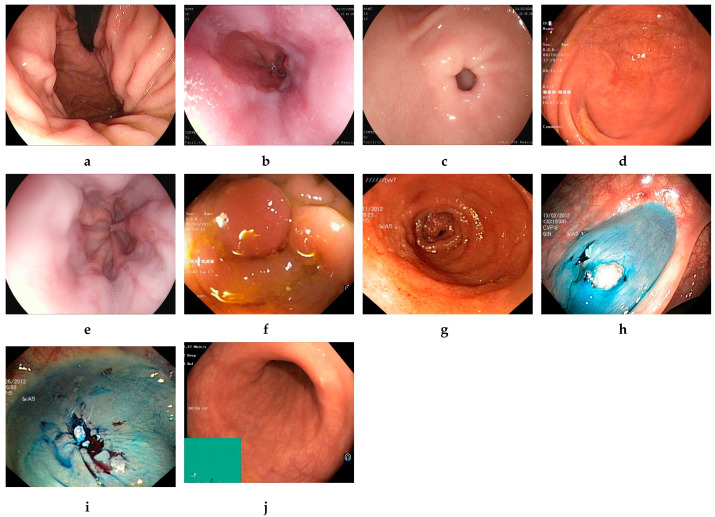
Instances of the endoscopic images of the HyperKvasir dataset: (**a**) retroflex stomach, (**b**) z-line, (**c**) normal pylorus, (**d**) normal-cecum, (**e**) esophagitis, (**f**) polyps, (**g**) ulcerative-colitis, (**h**) dyed lifted polyps, (**i**) dyed resection margins, and (**j**) mucosal view quality.

The additional dataset is known as HyperKvasir [48]. The photos and videos in this collection were obtained using conventional endoscopic apparatus from Olympus (Olympus Europe, Hamburg, Germany) and Pentax (Pentax Medical Europe, Hamburg, Germany) at a Norwegian hospital between 2008 and 2016. The collection comprises 10,662 annotated photos across 23 categories. The categories are unevenly distributed; hence, we selected only ten balanced categories to develop EndoNet. Four categories illustrate anatomical landmarks, three demonstrate pathological conditions, one illustrates mucosal view quality, and two pertain to lesion excision. The four distinct types of anatomical landmarks are the retroflex stomach, z-line, pylorus, and cecum. The three pathological conditions are esophagitis, polyps, and ulcerative colitis. The two categories related to lesion excision are dyed lifted polyps and dyed resection margins. Figure 2 displays examples of photos contained within the collection.

### 3.3. Proposed Framework

**Figure 3:**
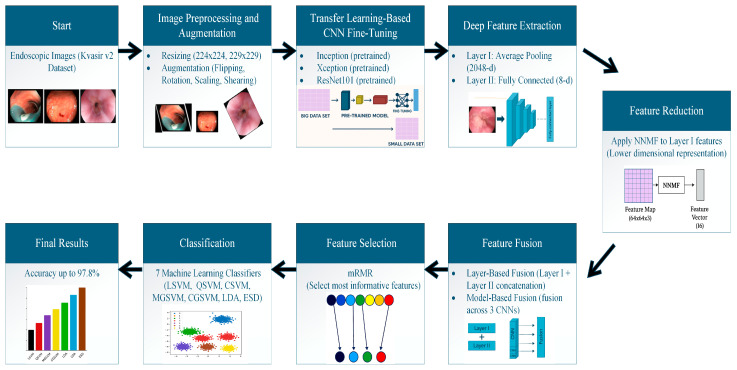
General summary of the steps of the suggested framework.

This study proposes a framework based on hybrid DL models and feature reduction and selection algorithms for classifying multiple GI diseases using endoscopic images. The proposed framework consists of five steps: Image preparation and Augmentation, DL Models Fine-Tuning and Retraining, Deep Feature Extraction and Reduction, Feature Fusion and Selection, and lastly, Multi-GI Disease Classification. Initially, endoscopic images are prepared by altering their dimensions to fit DL models. Afterward, these images are split into training and testing sets, followed by the application of various augmentation techniques on the training set. Next, multiple pre-trained CNNs are constructed, fine-tuned, and retrained on the endoscopic images. Deep features are then extracted from each CNN’s two different deep layers, namely layers I and II. Dimensions of layer I deep features are high, and thus the NNMF feature reduction approach is applied to lessen their dimension. Subsequently, deep features are integrated into two phases: layer-based CNN fusion and multi-CNN-based fusion. Following that, the mRMR feature selection method is applied to the multi-CNN-based integrated deep features to select the most influential features, thus reducing feature space dimensionality. Finally, seven ML classifiers are employed to classify the eight GI diseases. The steps of the proposed framework are shown in Figure 3.

#### 3.3.1. Image Preparation and Augmentation

Since each CNN accepts a specific input image dimension, the aspects of endoscopic images were modified to fit the acceptable input dimension of each CNN. For Inception and Xception, images were altered to be 229 × 229 × 3, while for ResNet-101 the dimensions were changed to 224 × 224 × 3. After that, the endoscopic Kvasir v2 and HyperKvasir datasets were split into 70–30% for training and testing sets, respectively. For training data, several augmentation techniques were applied to boost the number of training images and mitigate overfitting. These augmentation techniques were flipping, rotation, scaling, and shearing. The details of the ranges of the augmentation methods employed are shown in Table 1.

#### 3.3.2. DL Models Fine-Tuning and Re-Training

This step includes the construction of three pre-trained CNNs using transfer learning [49] including Inception, Xception, and ResNet101. The inclusion of Inception, Xception, and ResNet101 CNNs in the proposed framework is driven by their demonstrated efficacy in capturing intricate and varied visual patterns, crucial for the precise classification of various GI diseases in endoscopic images. Inception networks are recognized for their capacity to capture multi-scale features via parallel convolutional filters, rendering them appropriate for the analysis of diverse lesion sizes and textures [50]. Xception, an enhancement of Inception, utilizes depthwise separable convolutions to attain superior computational efficiency and enhanced feature extraction [51]. ResNet101, leveraging a deep residual learning construction, facilitates the effective training of extensive networks by alleviating the vanishing gradient issue, thereby enabling robust feature extraction from high-resolution medical images [52]. The integration of these architectures guarantees a thorough and distinct representation of GI anomalies across various imaging contexts. Transfer learning applied to these pre-trained CNNs means that these DL models were previously trained on huge image datasets such as ImageNet [53] in the same classification task but in different domains. Transfer learning is utilized by modifying the final output layers of the pre-trained CNN models to correspond with eight and ten different categories in the Kvasir v2 and HyperKvasir datasets, respectively. Furthermore, more settings within the CNN architectures were modified, with particular details addressed in subsequent sections. Subsequent to these adjustments, the three chosen pre-trained networks were retrained employing the Kvasir v2 and HyperKvasir endoscopic image datasets independently.

#### 3.3.3. Deep Feature Extraction and Reduction

CNNs generally initiate by recognizing low-level patterns in input images and subsequently acquire more intricate and meaningful features in deeper layers. To improve classification accuracy, transfer learning was applied to extract deep properties from two distinct layers of each CNN model. These two deeper layers chosen for feature extraction were the final average pooling layer (designated as Layer I) and the last fully connected (FC) layer (Layer II). Each of the Inception, Xception, and ResNet101 models produced a feature vector of length 2048 from Layer I for both datasets and a feature vector of length 8 and 10 from Layer II for the Kvasir v2 and HyperKvasir datasets, respectively. As noted, the dimensions of Layer I deep features are huge; therefore, the NNMF features reduction approach is adopted to lower their size. An ablation study was carried out to study the impact of different reduced feature sets on the classification performance.

#### 3.3.4. Feature Fusion and Selection

In this step, as mentioned earlier, deep features were combined into two phases: layer-based CNN fusion and multi-CNN-based fusion. Layer II deep features were concatenated with each CNN’s reduced Layer 1 deep features in the layer-based CNN fusion. This phase aimed to investigate whether fusing deep features from separate layers of a CNN can enhance diagnostic performance. In the multi-CNN-based fusion phase, the combined features of both layers of each CNN were merged for the three CNNs. This phase examined whether fusing features from multiple CNNs having distinct structures can boost classification accuracy. In this phase, the feature selection algorithm, namely MRMR, was applied to the merged features of the three CNNs to select the most significant features that enhance diagnostic performance and reduce classification complexity.

#### 3.3.5. Multi-GI Disease Classification

During the classification phase of endoscopic photographs, seven separate ML approaches were employed to differentiate among the eight unique categories found in the Kvasir v2 dataset. This encompasses the Ensemble Subspace Discriminant (ESD), Linear Discriminant Analysis (LDA), and five classifiers (Support Vector Machine (SVM)), each employing a distinct kernel function: cubic, linear, coarse Gaussian, quadratic, and medium Gaussian. Each classifier is engineered with a distinct computational strategy to enhance performance in multi-class image classification. Five-fold cross-validation was employed to ensure a thorough and unbiased evaluation. In each cycle, the aforementioned technique divides the dataset into five equal segments, using four for training and one for testing. The segments’ roles were rotated during the validation phase. This method ensures that each image is utilized for training and validation, leading to a thorough evaluation of each model’s efficacy. The comparative analysis of these classifiers underscores their ability to accurately differentiate the eight GI disease categories, providing significant insights into their utility for endoscopic image analysis in clinical diagnostics.

## 4. Experimental Setup and Results

In this section, we detail the experimental setup used to evaluate the performance of the proposed EndoNet framework, followed by a comprehensive presentation of the results obtained. The evaluation was conducted on the publicly available Kvasir v2 dataset, focusing on the classification accuracy of eight different gastrointestinal disease categories.

To assess the diagnostic performance of the model rigorously, a variety of standard statistical metrics were calculated. These include accuracy, sensitivity, specificity, precision, and F1-score, which collectively provide insights into different aspects of the classification quality. Furthermore, Receiver Operating Characteristic (ROC) curves and the corresponding AUC values were generated to evaluate the model’s ability to discriminate between different disease classes effectively. Details regarding ROC curve generation, threshold determination, and interpretation are described to provide a clear and robust framework for performance evaluation.

### 4.1. Performance Indicators

To thoroughly assess the classification efficacy of the presented EndoNet framework, several performance indicators were used, comprising accuracy, precision, sensitivity (recall), specificity, F1-score, and Matthews Correlation Coefficient (MCC). Accuracy quantifies the ratio of correctly identified cases to total predictions, offering a comprehensive assessment of model performance. Precision measures the proportion of true positive (TP) estimates to the overall amount of positive predictions, reflecting the model’s capacity to minimize false positives (FPs). Sensitivity (or recall) evaluates the proportion of accurately identified positive data points relative to all actual positives, indicating the model’s capacity to identify true instances of gastrointestinal disease. On the contrary, specificity quantifies the ratio of accurately identified true negatives (TN), illustrating the model’s proficiency in minimizing false positives. Sensitivity and harmonic average of precision, the F1 score, equilibrate false positives and false negatives (FN), rendering it especially advantageous in scenarios of class imbalance. Ultimately, the Matthews Correlation Coefficient (MCC) is a reliable metric that considers true and false positives and negatives, providing a more equitable evaluation of binary classification performance, particularly in scenarios with imbalanced class distribution. These metrics collectively offer a comprehensive and nuanced assessment of EndoNet’s efficacy in identifying GI diseases.

(6) $$Sensitivity=\frac{TP}{TP+FN}$$

(7) $$Specificity=\frac{TN}{TN+FP}$$

(8) $$Precision=\frac{TP}{TP+FP}$$

(9) $$MCC=\frac{TP\times TN-FP\times FN}{\sqrt{\left(TP+FP)(TP+FN)(TN+FP)(TN+FN\right)}}$$

(10) $$F\_1-score=\frac{2\times TP}{\left(2\times TP\right)+FP+FN}$$

(11) $$Accuracy=\frac{TP+TN}{TN+FP+FN+TP}$$

(12) $$AUC=\sum TP+\sum \frac{TN}{P+N}\;\;\;\;$$

### 4.2. Experimental Setup

The experimental configuration of the suggested EndoNet framework necessitated rigorous adjustment of critical hyperparameters to attain an optimal equilibrium between model efficacy and computational effectiveness. A mini-batch size of 10 was chosen to optimize memory usage during training while ensuring adequate gradient estimation precision. The training procedure was executed over 30 epochs, facilitating sufficient learning rounds for the model to reach convergence without overfitting. A learning rate of 0.001 was selected to achieve an equilibrium across convergence velocity and training stability. Validation was conducted every 500 iterations to assess the model’s improvement in learning and reduce overfitting. All additional hyperparameters were maintained at their default settings, guaranteeing uniformity and streamlining the experimental procedure. The learning process leveraged the Stochastic Gradient Descent with Momentum (SGDM) optimizer, which improves convergence by integrating momentum to mitigate oscillations and expedite progression through shallow areas in the loss landscape. The selection of hyperparameters enhanced the model’s strong performance across all evaluation metrics, affirming the efficacy and dependability of the proposed CAD framework. All experiments were conducted using MATLAB R2022b. A system with an Intel(R) Core (TM) i7-10750H CPU operating at 2.6 MHz was employed for this research. It also included an NVIDIA GeForce GTX 1660 graphics card with 6 GB of RAM and a 64-bit operating system.

### 4.3. Results

This section provides a detailed presentation of the experimental findings related to the suggested EndoNet framework’s classification performance. The applications were carried out on the Kvasir v2 endoscopic image dataset, and the effects of different feature extraction, reduction, fusion, and selection strategies on classification accuracy were systematically evaluated. The feature vectors obtained at each stage were tested with seven different ML classifiers: linear SVM (LSVM), quadratic SVM (QSVM), cubic SVM (CSVM), medium Gaussian SVM (MGSVM), coarse Gaussian SVM (CGSVM), LDA, and ESD. The evaluations were carried out using a five-fold cross-validation method. The results show the effect of using features extracted from different layers separately and together, the combination of multiple CNN architectures, the contribution of the feature reduction process with NNMF, and the classification performance of dimensionally reduced but effective features obtained with mRMR feature selection. The findings of each experimental structure are presented in detail below, respectively, with table references.

#### 4.3.1. Multi-Layer Feature Extraction Results

In the deep feature extraction process, the 2048-dimensional deep features obtained from the average pooling layer (Layer I) of each CNN model were used separately to perform classification. These features were directly given to seven different ML algorithms without any dimension reduction process. Table 2 shows the classification performance of these raw deep features. According to the results, in general, the ResNet101 model provided the highest accuracy in all classifiers, and it was seen that this model worked in harmony with the LSVM (96.5%), QSVM (96.3%), and ESD (96.1%) algorithms. The Xception model also produced very successful results, especially in the QSVM and CSVM classifiers; it stood out with 96.2% accuracy. The accuracy of the Inception model was slightly lower than that of these two models, and the highest accuracy rate was 96.1% with QSVM. These findings show that each CNN architecture has the power to capture different visual representations and that the deep features obtained from the pooling layer provide very strong representations for classification. It is understood that especially the deep layers of ResNet101 contribute significantly to the overall accuracy by capturing more distinct features.

Evaluation of the HyperKvasir dataset showed that all three CNN models showed strong classification performance, with all classifiers performing above an accuracy of 96.5%. Of the CNN models, Xception produced superior model performance results as it produced the top classification accuracies of 97.5% using LSVM, 97.4% using QSVM, and 97.3% using CSVM classifiers. This shows that Xception is the best possible model for endoscopic images among the existing models, as it demonstrates performance with classification accuracy of endoscopic images to extract discriminative features from images. It is likely that the depth separable convolution structure of the Xception model is why the model is able to use a limited number of convolutional parameters and still show performance greater than Inception. ResNet101 showed stable performance in the analysis with accuracy values from 96.2% to 96.9%. This shows that ResNet101 is a reliable model for extracting higher-level semantic features of medical images. Overall, Inception was slightly more challenging to classify than Xception, with the maximum accuracy being 96.9% using the LSVM and QSVM models. The high level of performance in the CNN models on the HyperKvasir dataset suggests that using deep features from the pooling layer contains useful information for classification and demonstrates that transfer learning can use pre-trained models to work in the context of classification of the specific domain of gastrointestinal disease.

In the next experiment, the classification was made by directly using the 8-dimensional features extracted from the last FC layer (Layer II) of each CNN model. Table 3 shows the accuracy values obtained for these features with seven different ML classifiers. When the results are examined, the Xception model showed the best performance with the features obtained from the FC layer. In particular, 96.4% accuracy was obtained with LSVM, 96.1% with CGSVM, and 95.9% with LDA. The ResNet101 model showed similarly stable performance, giving 96.0% results with MGSVM and CGSVM, and 95.0–96.2% with other classifiers. The Inception model, on the other hand, showed lower performance compared with the other two models, and a relatively weaker accuracy rate of 94.7% was obtained with the CSVM classifier. These findings show that FC layers provide more concise but meaningful representations and that these features contribute significantly to the classification performance, especially in more optimized architectures such as Xception. It is also seen that low-dimensional features provide effective classification performance without the need for dimensionality reduction.

The analysis of the HyperKvasir dataset yields the following findings. In the majority of classifiers, the Xception model achieved the highest accuracy (97.7% accuracy for LSVM, 97.5% accuracy for CGSVM, and 97.3% accuracy for QSVM), which underlines its ability to learn discriminative features effectively using the endoscopic images. The ResNet101 was consistently a strong performer and achieved accuracy values ranging from 96.1 to 97.1%, as well as a particularly high performance with LSVM (97.1%) and MGSVM (96.9%). The Inception model had a less consistent performance compared with the other models, but still approached an accuracy of 97% with the highest value of 97.0% with LSVM. These results suggest historical features in the FC layers are a viable means of machine cutting classification regardless of further feature reduction strategies. The performance of the Xception model on HyperKvasir demonstrates it has the potential to be a strong candidate for practical multi-class GI disease diagnoses, with significant potential for enhanced automated, endoscopic image analysis in a clinical application.

#### 4.3.2. Dimensionality Reduction Results

In this experimental stage, dimensionality reduction was performed by applying the NNMF method to 2048-dimensional features extracted from the Layer I (pooling) layer of each CNN model. The aim was to evaluate whether similar or better classification performance can be achieved with fewer features. The effect of altering the quantity of NNMF features on the classification performance was investigated using an ablation study. Table 4 shows the accuracy results obtained with feature numbers varying between 10 and 50. In general, it is seen that the accuracy rates increase as the number of features increases. The best results for the Inception model were obtained with LSVM (95.5%), QSVM (95.4%), and ESD (94.7%) in the 50-feature case. However, some classifiers, such as ESD and LDA, experienced a more significant performance loss in low-dimensional representations. For example, ESD provided only 90.9% accuracy with 10 features. Res-Net101 model showed stable performance, especially in the 40 and 50 feature cases with LSVM (95.6%), CSVM (94.5%), and other classifiers. It was observed that this model gives very efficient results with NNMF, especially in the range of 30–50 features. Similarly, the Xception model produced accuracy values reaching up to 95.3% with QSVM and CSVM in the number of features between 30 and 50. Table 4 shows that the dimensionality reduction process performed with NNMF can largely preserve the classification performance and even improve it for some classifiers. In particular, making high-dimensional deep features more compact and interpretable increases the generalizability and processing efficiency of the system.

Table 5 summarizes the classification results achieved with the NNMF-based dimensionality reduction, on the pooled layer embedding features from each CNN model (Inception, ResNet101, and Xception) with the HyperKvasir dataset. The results suggest the classification performance is strong even with much lower dimensionality after dimensions were reduced from 2048 to a minor number, which varied from 10 to 50 features. Inception obtained its highest accuracy of 96.7% using LSVM and QSVM with 50 features, which indicates NNMF preserved discriminative information while dimensionality was condensed. ResNet101 offered stable performance over the number of features tested, revealing its best performance (96.2% using LSVM with 40 features), indicating that the learned deep features can be compressed without loss to classification accuracy. While Xception offered the best results regardless of dimensionality reduction achieved, it produced an impressive best accuracy of 97.1% with LSVM at 30 features, showing that it produces superior features with extreme dimensionality reduction, while most classifiers performed better with 50 features. Overall, the findings demonstrate NNMF is an effective dimensionality reduction technique that preserves high classification accuracy using condensed feature representations, particularly with the Xception model on the HyperKvasir dataset.

#### 4.3.3. Deep Layer Fusion Level Results

In the next experiment of the proposed EndoNet, a richer attribute representation was created by combining the Layer I (Pooling) and Layer II (FC) deep properties of each CNN model (fused). The properties extracted from the pooling layer were reduced from 2048 to 40 using the NNMF method, combined with the 8-dimensional properties obtained from the FC layer, creating a 48-dimensional unified feature vector in total. The accuracies achieved using the combined feature vectors with seven different classifiers for each CNN model, along with comparisons to the previous cases, are presented in Table 6.

The highest accuracy value for the Inception model was obtained when the combined features were used with LSVM (96.7%) and QSVM (96.3%) classifiers. This result is above the accuracies provided by FC (96.1%, 95.9%) or Pool layers (96.0%, 96.1%) alone and shows that multi-layer feature fusion increases the classification success. On the other hand, the ResNet101 model achieved very high accuracies with the combined features in LSVM (96.7%), QSVM (96.4%), and ESD (96.0%) classifiers. Especially, in this model, the use of both FC and NNMF applied to the Pool features together provided more balanced and stable classification results. Similarly, the Xception model achieved the highest accuracy in combined features with QSVM (96.3%) and LSVM (96.5%). According to the results obtained with FC (96.0%,96.4%) or Pool (96.2%,96.2%) layers alone, the combination increased the classification performance. These findings show that the attributes obtained from different layers of CNN architectures are complementary to each other, and combining these representations offers a more solid classification basis. In addition, it was seen that with NNMF, reduced-dimensional features can be integrated with FC features efficiently without causing information loss.

Table 7 presents the classification results for the HyperKvasir dataset that show the efficacy of concatenating the deep features extracted from both the pooling (Layer I) and FC (Layer II) layers of three CNN models (Inception, ResNet101, and Xception), implementing NNMF for both dimensionality reduction and feature concatenation. The combined features (Pool_NNMF + FC), for each model, resulted in a higher accuracy than either the Pool features (97.0–97.1%), Pool_NNMF (96.9%), or FC features (97.0%). This is an indication of the synergistic benefits of layer-pooling representation and the use of multi-layer feature representations. The Inception model achieved a maximum accuracy of 97.3% with both LSVM and QSVM classifiers using the 60-dimensional combined features. The Pool (96.8%, 96.9%), Pool_NNMF (96.7%, 96.4%), and FC features (97.0%, 96.9%) accuracies were lower than the combined Pool_NNMF + FC values (97.3%,97.3%), The ResNet101 model yielded a maximum accuracy of 97.6% with LSVM classifier using the combined 50-dimensional features, outperforming Pool (96.9%), Pool_NNMF (96.2%) and FC (97.1%) features. The Xception model achieved 97.6% accuracy with the QSVM classifier on the combined (60-dimensional) features compared with Pool (97.4%), Pool_NNMF (96.7%), and FC (97.3%). This illustrates that concatenating NNMF-pooling features with FC features achieves improved classification performance by eliminating overlapping feature representation while maintaining categorized discriminative information. Across all classifiers, LSVM and QSVM always produced the highest accuracies, highlighting the potential for multi-class endoscopic image classification when the models were trained on compact, fused feature representations. The increased performance with combined features indicated the benefits of the multi-layer fusion approach to incorporate both higher-level semantic information and lower-level class-specific information, further supporting the key element of combining efforts in order to create the proposed EndoNet framework for accurate and robust classification of gastrointestinal diseases.

#### 4.3.4. Deep Networks Fusion Level Results

In the following experiment of EndoNet, all deep features obtained from Inception, Xception, and ResNet101 models were combined (including pooling and FC), and a combined feature set of 144 dimensions was created. The mRMR method was applied to this feature set, and the most informative ones were selected for different feature numbers, classification was performed with these features. Table 8 presents the accuracy results obtained for feature numbers ranging from 10 to 100 and the case where all features were used together. In the case where all features were used (144 features), the accuracy rates were quite high, with LSVM 97.7%, QSVM and CSVM 97.6%, MGSVM 97.3%, CGSVM 97.5%, LDA 97.1%, and ESD 97.0% accuracy. This shows that the features from multiple CNN architectures together provide a strong representation. When the number of features selected with the mRMR method is 70, the highest accuracy value in the study, 97.8%, was obtained with LSVM. Similarly, with 90 features, 97.7% accuracy was obtained with QSVM and CGSVM classifiers, and 97.2% with ESD, and very successful results were achieved. This shows that selecting the most appropriate features of multiple model representations can further increase the classification performance and that the system can be made more efficient by purifying it from redundant data. As a result, feature selection with mRMR both increases the classification accuracy and reduces the computational load, contributing to the system being faster and more scalable. The accuracies obtained with the features selected in the range of 70–90 are equal to or higher than the results obtained with the full feature set.

The results reported in Table 9 demonstrate the performance of the proposed EndoNet framework for using the HyperKvasir dataset to classify gastrointestinal diseases using the mRMR feature selection algorithm on the combined deep features of all CNN models. The classification performance of the proposed EndoNet framework was evaluated using seven ML classifiers, demonstrating that the LSVM classifier was most effective, achieving an accuracy of 98.4% when using 100 selected features. The second most successful classifiers, CGSVM and CSVM, were close behind at 98.1% and 98.1% accuracy using 40 and 70, respectively. The results from these classifiers further identified the superiority in performance of SVM-based classifiers. Even with fewer features (e.g., 100 features using LSVM or 40 using QSVM), the accuracy of the proposed framework demonstrated effectiveness (98.4% and 98.3%, respectively), suggesting that mRMR was able to reduce dimensionality while maintaining important discriminative characteristics. We also see potential through the established predictability level with the other classifiers and feature selection strategies, like ESD and LDA, indicating generalizability to all fused deep features because of the characteristics of the related fused deep features.

Finally, following the application of the mRMR algorithm in feature selection, detailed performance metrics of seven different ML classifiers were calculated using the most significant selected attributes. Table 10 shows the sensitivity, specificity, precision, F1-score, and MCC (Matthews Correlation Coefficient) values for each classifier. The results reveal that the LSVM classifier in particular achieved the highest overall success (sensitivity: 0.9779, specificity: 0.9969, precision: 0.9781, F1-Score: 0.9779, and MCC: 0.9749). This performance shows that LSVM is the algorithm that provides the most balanced and reliable classification with selected features.

QSVM and CGSVM classifiers also gave very close results to LSVM. For example, QSVM has a very high MCC value of 0.9732. Although MGSVM and CSVM gave successful results in the range of 0.9745–0.9749 in terms of sensitivity, they did not achieve a consistent overall success as LSVM. More traditional classifiers such as LDA and ESD also showed competitive performances, especially in terms of MCC and F1-Score (e.g., MCC for ESD: 0.9689). However, more powerful SVM variants, such as LSVM and QSVM, gave more efficient results with the features selected using mRMR. Table 10 also illustrates the performance metrics that demonstrate the efficacy of the proposed EndoNet architecture in attaining high diagnostic accuracy on the HyperKvasir dataset, especially when employing features picked via the mRMR technique. Of the seven ML classifiers assessed, the LSVM classifier exhibited the highest overall efficacy, attaining a sensitivity of 0.983, specificity of 0.998, precision of 0.984, F1-score of 0.983, and an MCC of 0.981, signifying its exceptional capability to differentiate among the ten distinct gastrointestinal disease categories with minimal false positives and false negatives. QSVM and CSVM classifiers achieved comparable outcomes, closely trailing LSVM in sensitivity (0.982 for both) while sustaining high specificity (0.998) and MCC values (0.980 for both), indicating their efficacy in managing multi-class classification tasks with diminished feature sets. The MGSVM and CGSVM classifiers, albeit marginally less consistent than LSVM, exhibited robust performance, with sensitivity values of 0.980 and 0.979, respectively, and MCC values above 0.977, thereby reinforcing the framework’s dependability in precise disease categorization. Conventional classifiers like LDA and ESD produced notable outcomes, with LDA attaining a sensitivity of 0.981 and ESD achieving 0.980, indicating that even rudimentary models can greatly benefit from proficient feature selection.

**Figure 4:**
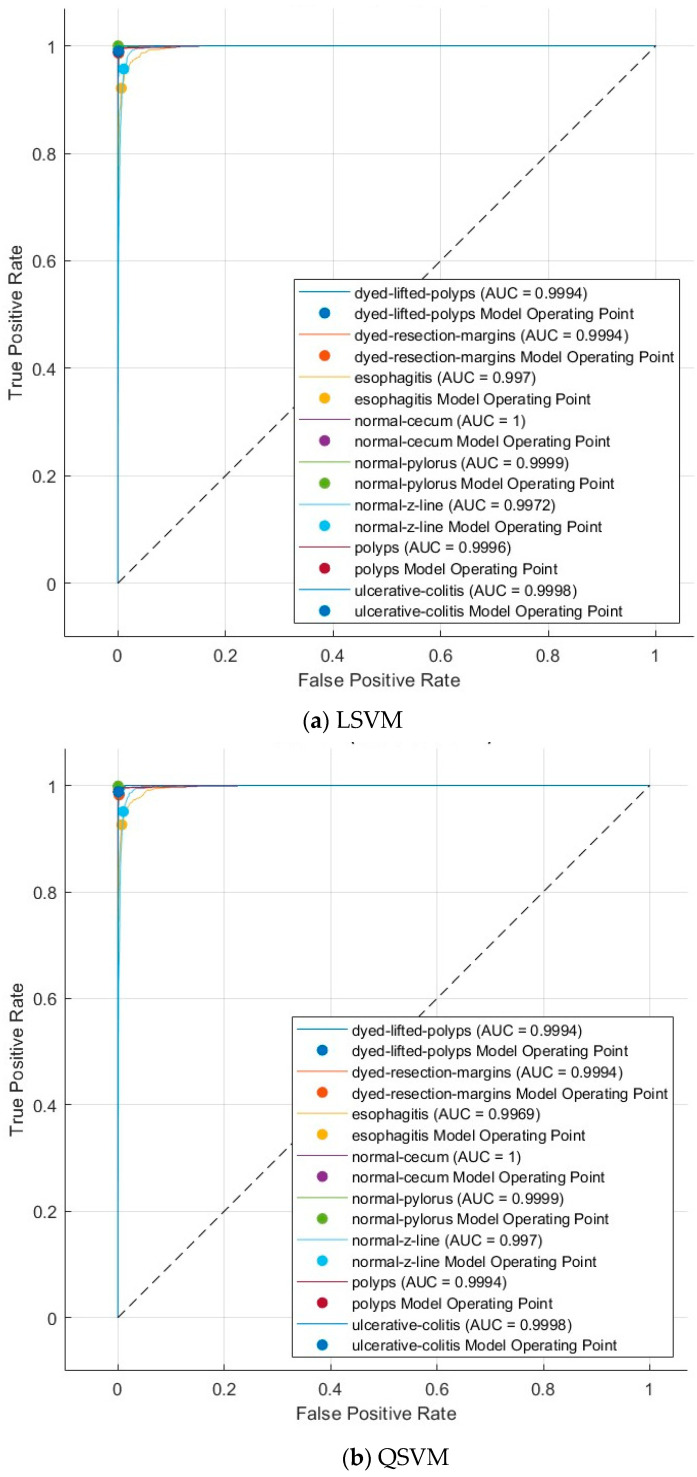
ROC curves of LSVM (70 features) and QSVM (90 features) trained with Kvasir v2 dataset.

**Figure 5:**
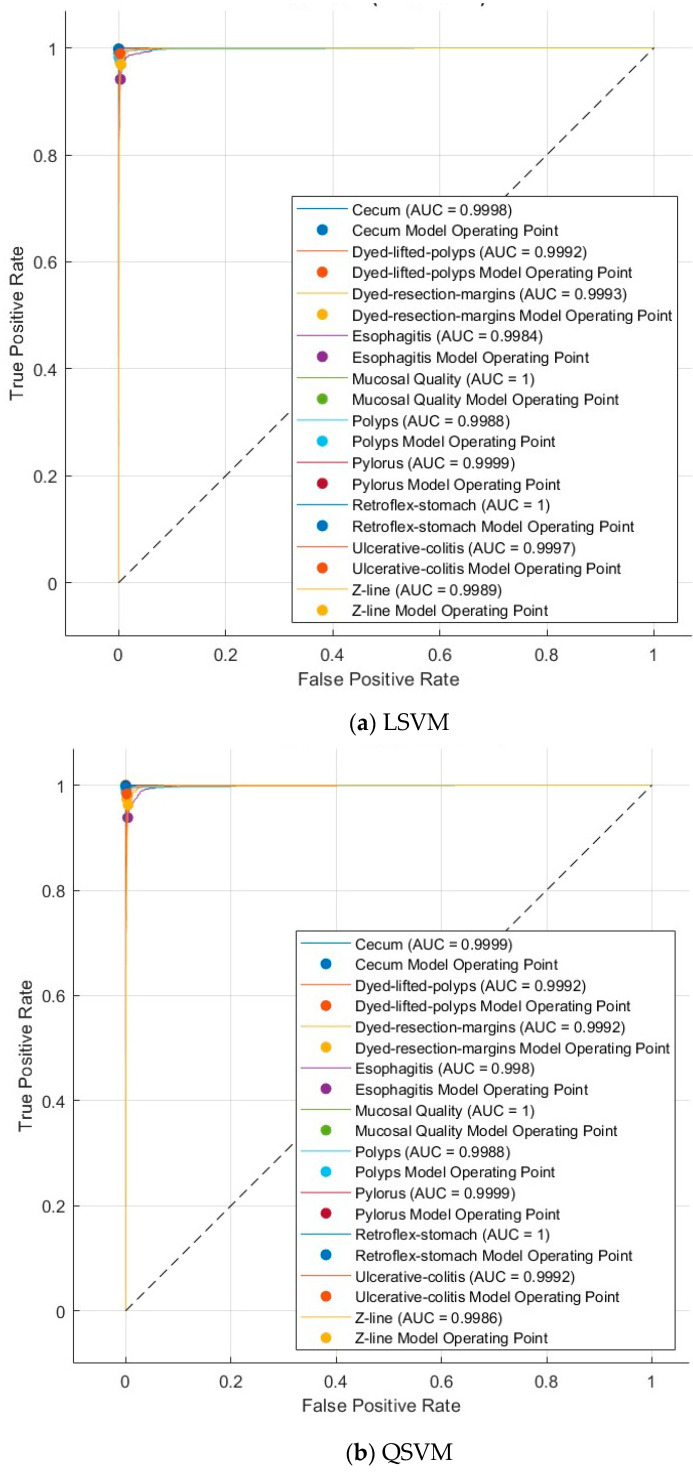
ROC curves of LSVM (100 features) and QSVM (40 features) trained with the HyperKvasir dataset.

**Figure 6:**
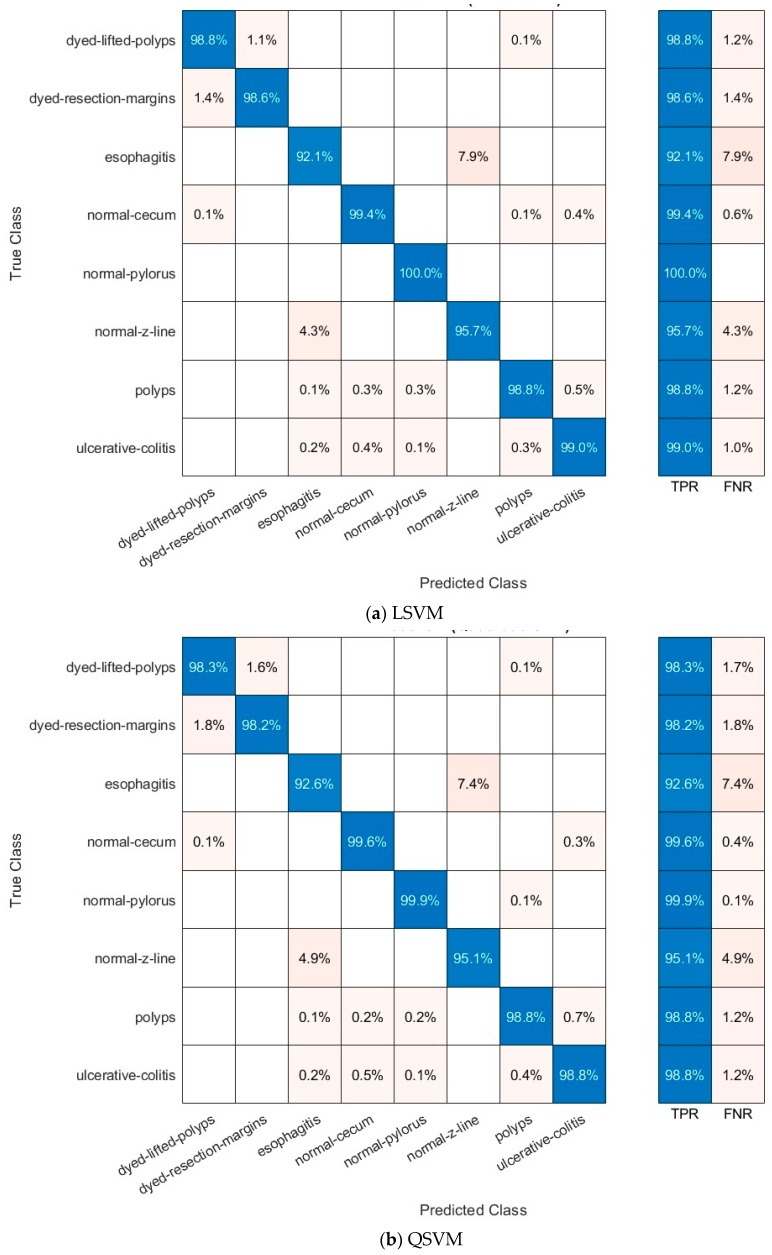
Confusion matrices of LSVM (70 features) and QSVM (90 features) trained with the Kvasir v2 dataset.

**Figure 7:**
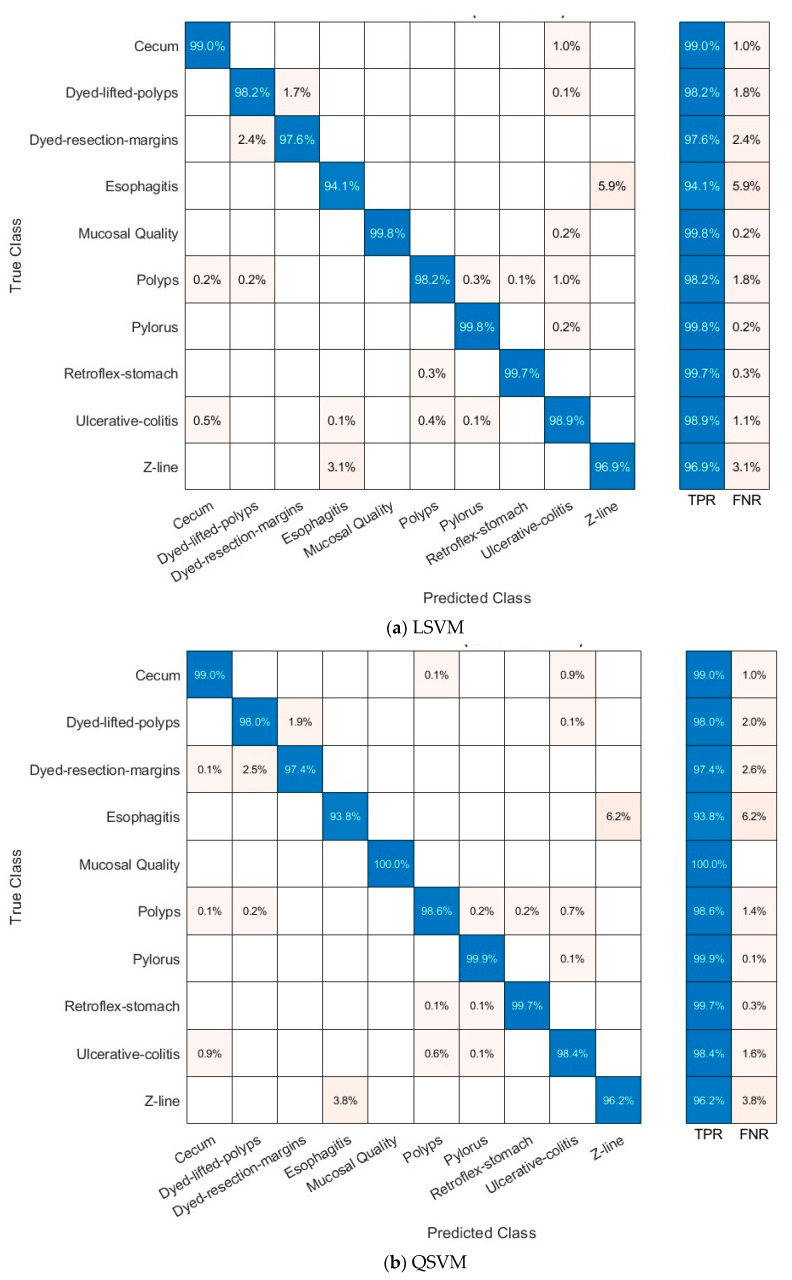
Confusion matrices of LSVM (100 features) and QSVM (40 features) trained with the HyperKvasir dataset.

Figure 4 and Figure 5 present the ROC curves for the Kvasir v2 dataset, and Figure 6 and Figure 7 show confusion matrices of the LSVM and QSVM models in order to visually evaluate the classification success of the proposed framework. The ROC curves in Figure 4 and Figure 5 show the verification accuracy of the classifications performed using the LSVM classifier with 70 and 100 selected features and the QSVM classifier with 90 and 40 features for the Kvasir v2 and HyperKvasir datasets, respectively. The width of the AUC reveals that both models have a high power of distinctiveness. Especially in the LSVM model, the fact that AUC values are quite high across classes shows that a strong classification performance can be achieved even with fewer features. The confusion matrices presented in Figure 6 and Figure 7 reveal the classification accuracy of both models in more detail. While the LSVM model correctly classifies most of the eight and ten different gastrointestinal diseases of the Kvasir v2 and HyperKvasir datasets, respectively, it only has limited error rates in some classes. Furthermore, the QSVM model achieved high correct classification rates for both datasets, especially in examples where the distinction between classes was more difficult. When these two images are evaluated together, it is clearly seen that the few but effective features selected with the mRMR method can provide high accuracy both overall and on a class basis.

In general, these findings reveal that LSVM is the most reliable classifier in the proposed system, not only in terms of accuracy rate but also in terms of sensitivity, specificity, and balance measurements. Thanks to the mRMR feature selection, a classification structure with both high information density and strong generalizability was created.

## 5. Discussion

Classification of GI diseases has been a remarkable research area in recent years in terms of developing computerized diagnostic systems. The EndoNet framework proposed in this study offers a multi-layered and multi-stage approach by combining features extracted from different deep layers of multiple CNN architectures, dimensionality reduction with NNMF, and selecting meaningful features with the mRMR method and testing them with different classifiers.

The use of multi-layer feature fusion enhances the interpretability of the proposed framework by incorporating both low-level and high-level image features. Shallow layers typically capture fine-grained texture, edge, and color information, while deeper layers represent more abstract, semantic content such as lesion structure or disease type. By combining these layers, the model generates a richer and more semantically diverse feature space that aligns more closely with the hierarchical reasoning process used by clinicians during image assessment. This fusion facilitates more explainable predictions and potentially improves clinical trust in automated decision-support systems.

### 5.1. State of the Art Comparison

Important studies conducted in the field of GI disease classification, explained in Section 2, are summarized comparatively in Table 11 together with the methods used and the success rates achieved. This table systematically presents the variety and performance differences of current approaches based on the Kvasir datasets. In general, ensemble approaches, transfer learning with pretrained CNN-based methods, and hybrid methods based on attribute selection are widely preferred in the literature. In particular, advanced techniques such as saliency maps, attention mechanisms, and optimization algorithms have been effective in increasing classification accuracy. Segmentation-oriented studies also occupy an important place. However, multi-stage structures such as the systematic merging of deep features extracted from different layers of the same CNN model and the fusion of complementary representations of different CNN architectures have been considered in a limited number of studies. In this context, the proposed EndoNet framework offers a unique approach that fills the gaps in the literature by integrating cross-layer and cross-model feature fusion with dimensionality reduction and feature selection.

Although Sivari et al. [30] achieved high accuracies of 98.42% with the stacking ensemble structure, only a limited number of CNN models and statistical evaluations were performed in their work. The study did not employ feature reduction or selection methods to reduce dimensionality. However, the EndoNet framework systematically addresses strategies that are rarely applied in the literature, such as inter-layer feature fusion. Gunasekaran et al. [31] reported 92.96% accuracy by combining DenseNet201, InceptionV3, and ResNet50 models with average weights but did not include deep feature fusion and feature selection processes at the layer level.

In the study by Khan et al. [33], structures such as segmentation, saliency maps, and MobileNet-V2 were used. However, it is seen that the proposed structures for classification are based on limited feature representation and have only been tested with certain datasets. EndoNet, on the other hand, has created a richer feature set by combining complementary representations of three different CNN architectures on a layer-by-layer basis. On the other hand, studies like [39] and [43] employed individual CNN models, ignoring the benefits of integrating the advantages of multi-CNN architectures.

El-Ghany et al. [35] tested several transfer learning techniques on the Kvasir-v2 amd HyperKvasir dataset, but the classification process did not include feature selection and fusion steps. On the other hand, although the study conducted by Khan et al. [38] fused deep features of two novel architectures, including SC-DSAN and CNN-GRU, they achieved an accuracy of 95.10%, which is lower than that attained using EndoNet (97.8%). Similarly, Janutėnas and Šešok [37] achieved 6.3% accuracy improvement by improving attention mechanisms with EfficientNet-B7, reaching 95.25% accuracy, but did not address multi-CNN fusion at the feature level. Moreover, the study [36] fused three CNNs and reached an accuracy of 91.00% without feature selection or reduction. This performance is much lower than that achieved using EndoNet, which employed mRMR and NNMR feature selection and reduction methods.

Kamble et al. [39] and Mohapatra et al. [40] generally worked with a single model or limited features in CNN-based structures and did not apply systematic dimensionality reduction or selection strategies. EndoNet, on the other hand, reports one of the most successful results in this field by showing that the best 70 features selected from 144 features give 97.8% accuracy with LSVM. In addition, EndoNet has outperformed other studies [48,54,55] trained on the HyperKvasir dataset.

As a result, unlike previous studies, the EndoNet framework systematically combines features from multiple CNN architectures on a layer-by-layer basis. It reduces the number of features with NNMF and selects information-dense features with mRMR. Then, it tests these features on seven different ML algorithms. This framework combines critical steps such as inter-layer fusion, dimensionality reduction, and classification with selected features, which are missing in most studies in the literature, and lays the foundations for an advanced and holistic AI-based decision support system for GI diagnosis.

### 5.2. Limitations and Future Directions

Despite the remarkable performance and high classification accuracy demonstrated by the proposed EndoNet system, multiple drawbacks must be recognized. The study employed the Kvasir v2 and HyperKvasir datasets, publicly accessible and varied collections of gastrointestinal endoscopic images; however, actual clinical environments frequently exhibit more disparities in image quality, lighting conditions, and anatomical differences among patients. The framework’s robustness and generalizability should be further validated using larger and more diverse datasets obtained from various healthcare institutions. Furthermore, dependence on a singular dataset may unintentionally introduce data-specific biases, potentially constraining the model’s efficacy when applied in wider clinical settings.

A further limitation is the absence of end-to-end training for the complete pipeline. The framework comprises several sequential stages—feature extraction, reduction, selection, and classification—each optimized independently. This modular design improves comprehension and flexibility but may lead to inferior global performance relative to an end-to-end DL architecture capable of jointly optimizing all components. Furthermore, while mRMR and NNMF are proficient in feature selection and dimensionality reduction, investigating more sophisticated or adaptive feature selection techniques may enhance the model’s performance and efficiency.

The clarity of the model’s decision-making requires improvement. The feature selection process enhances transparency; however, clinicians frequently necessitate visual clarifications or saliency maps to establish trust in AI-driven diagnostic tools. The incorporation of explainable AI (XAI) methodologies, such as Grad-CAM or SHAP, would yield intuitive visual representations of the model’s rationale and enhance its integration into clinical workflows.

Furthermore, the current study’s lack of direct clinical input is a constraint of this study, and this is clearly mentioned in the discussion section. We are actively looking to work with gastroenterologists and other medical professionals to drive this research’s subsequent phases. We assert that this partnership will be crucial for converting our findings into practical clinical applications, such as incorporation into endoscopic systems or utilisation in clinical decision support tools.

Although the classification accuracy of the presented EndoNet architecture is encouraging, it is crucial to acknowledge the constraints regarding its clinical usefulness. This study’s datasets encompass many classes, including Z-line, pylorus, and dyed resection margins, which, although anatomically or technically pertinent, do not immediately impact diagnostic or treatment decisions. The claimed high classification accuracy, while theoretically valid, needs to be approached with care regarding clinical application.

To augment the therapeutic utility of the proposed approach, further research will concentrate on endoscopic gastrointestinal tract datasets that highlight pathological significance, especially those with biopsy-confirmed neoplastic lesions, early-stage gastrointestinal malignancies, or bleeding lesions. Including each of these lesion categories will facilitate the assessment of both classification accuracy and the model’s capacity to assist in clinical decision making, risk stratification, and treatment planning. Incorporating pathology confirmation as the fundamental standard will allow for more stringent clinical validation and adherence to regulations. The transition to clinically actionable targets is a crucial subsequent phase in evolving EndoNet from a proof-of-concept to a functional element of AI-driven decision support systems in gastroenterology.

Future studies ought to concentrate on creating a comprehensive, end-to-end trainable model that integrates feature learning, selection, and classification within a singular architecture. The incorporation of temporal data from video capsule endoscopy, as opposed to exclusively employing static images, could enhance the diagnostic context and augment classification precision. Cross-domain adaptation methods and semi-supervised learning approaches should be explored to improve the model’s adaptability in data-scarce or label-restricted contexts. Cooperation with medical professionals for the real-time validation and implementation of the EndoNet framework in clinical decision-making systems is essential for transferring this study into actual healthcare applications.

## 6. Conclusions

In this study, a comprehensive and multi-stage DL-based framework called EndoNet has been proposed for the classification of multiple GI diseases using endoscopic images. The proposed framework includes the steps of preprocessing and augmenting the images belonging to the Kvasir v2 and HyperKvasir datasets, retraining three different pre-trained CNN models (Inception, Xception, and ResNet101) with fine-tuning, extracting deep features from two different layers of each model, reducing the dimensionality of these features with NNMF, a two-stage feature fusion process both between layers and between models, then selecting the most meaningful features with mRMR, and finally classifying with seven different ML algorithms. As a result of the experiments carried out, it was seen that the proposed system has reached quite high accuracy values. In particular, 97.8% and 98.4% classification accuracies were achieved with the LSVM classifier for the Kvasir v2 and HyperKvasir datasets, respectively. High performances were also achieved with other classifiers (QSVM, CSVM, ESD, etc.), which shows that especially combined and selected, features increase the classification power. Compared with single-layer or single model-based methods, the use of features obtained from multiple layers and architectures together provides a more explanatory and discriminative feature representation.

Compared with the current literature summarized in Table 11, the EndoNet model yielded more successful results compared with many studies that use only direct CNN models, do not make attribute selection, or work with a limited model structure. This approach, in which DL and traditional ML methods are used together, offers significant advantages in terms of interpretability, computational efficiency, and generalizability, as well as high accuracy. As a result, it is thought that the proposed system can provide a solid foundation for future AI-supported clinical decision-support systems. In future studies, investigation of attention mechanisms, explainable AI (XAI) methods, and integration with real-time endoscopy systems can further strengthen the applicability of this method in the clinical field.

## Figures and Tables

**Table 1:** Augmentation methods range of values used to augment training images.

Augmentation Method	Range of Augmentation
Flipping in X and Y orientations	−40 to 40
Rotation in X and Y orientations	50% probability
Scaling in X and Y orientations	0.5 to 2
Shearing in X and Y orientations	−50 to 50

**Table 2:** Classification results (%) obtained with Layer I (pooling) features for Kvasir v2 and HyperKvasir datasets.

Model	LSVM	QSVM	CSVM	MGSVM	CGSVM	LDA	ESD
Kvasir v2 Dataset							
Inception	96.0	96.1	95.8	95.7	95.6	95.6	95.5
ResNet101	96.5	96.3	96.3	95.7	95.8	95.8	96.1
Xception	96.2	96.2	96.2	95.7	96.0	95.5	95.8
HyperKvasir Dataset							
Inception	96.8	96.9	96.9	96.7	96.5	96.3	96.6
ResNet101	96.9	96.8	96.8	95.9	96.2	96.3	96.6
Xception	97.5	97.4	97.3	97.0	97.3	97.1	97.2

**Table 3:** Classification results (%) obtained with Layer II (Fully Connected) features.

Model	LSVM	QSVM	CSVM	MGSVM	CGSVM	LDA	ESD
Kvasir v2 Dataset							
Inception	96.1	95.9	94.7	95.7	95.8	95.3	95.0
ResNet101	96.2	95.9	95.2	96.0	96.0	95.3	95.0
Xception	96.4	96.0	95.2	96.0	96.1	95.9	95.5
HyperKvasir Dataset							
Inception	97.0	96.9	96.0	96.8	96.3	96.1	95.7
ResNet101	97.1	96.8	96.1	96.9	96.6	96.2	96.8
Xception	97.7	97.3	96.4	97.3	97.5	97.6	97.5

**Table 4:** Classification results (%) obtained by applying NNMF to ooling features for the Kvasir v2 dataset.

# Features	LSVM	QSVM	CSVM	MGSVM	CGSVM	LDA	ESD
Inception							
10	95.0	94.8	94.0	94.7	94.3	92.1	90.9
20	95.6	95.2	94.4	95.0	95.4	94.3	93.8
30	95.4	95.3	94.6	95.1	95.1	94.5	94.4
40	95.8	95.2	94.9	95.1	95.1	94.2	93.9
50	95.5	95.4	94.8	95.0	95.2	94.7	94.7
ResNet101							
10	94.6	94.3	93.5	94.1	93.9	92.0	90.4
20	95.2	94.4	93.5	94.4	94.6	93.0	92.5
30	95.2	95.1	94.2	94.7	95.0	94.1	94.0
40	95.6	95.0	94.5	94.6	95.1	94.3	94.0
50	95.2	95.2	94.7	94.7	94.9	94.4	94.1
Xception							
10	95.1	94.7	93.5	94.5	94.7	93.9	93.9
20	95.2	94.7	93.8	94.5	95.1	94.3	93.9
30	95.3	95.0	94.1	94.5	95.0	94.8	94.2
40	95.3	95.3	94.5	94.8	95.1	94.5	94.1
50	95.2	95.1	94.4	94.6	95.0	94.5	94.6

**Table 5:** Classification results (%) obtained by applying NNMF to Pooling features for the HyperKvasir dataset.

# Features	LSVM	QSVM	CSVM	MGSVM	CGSVM	LDA	ESD
Inception							
10	95.3	95.2	94.4	95.4	95.0	91.3	90.8
20	96.7	96.2	95.6	96.1	96.3	94.8	95.1
30	96.6	96.2	95.7	96.3	96.2	96.0	95.7
40	96.5	96.3	95.9	95.9	96.5	96.1	95.8
50	96.7	96.4	95.8	96.1	96.6	96.0	95.8
ResNet101							
10	93.7	93.4	92.6	93.2	92.8	88.5	87.1
20	96.0	95.6	94.9	94.9	95.1	94.3	93.7
30	96.1	95.7	94.9	94.5	95.2	94.6	93.5
40	96.2	95.9	95.5	94.8	95.6	94.8	94.0
50	96.1	95.9	95.2	94.3	95.0	95.3	94.5
Xception							
10	96.8	96.3	95.5	96.1	96.5	96.3	96.0
20	97.0	96.5	95.6	95.9	96.6	96.3	95.7
30	97.1	96.5	96.0	95.8	96.6	96.7	96.3
40	96.9	96.4	95.8	95.6	96.5	96.8	96.5
50	96.9	96.7	95.8	95.8	96.5	97.0	96.6

**Table 6:** Classification accuracies (%) for the Kvasir v2 dataset after combining both FC and Pooling features after applying NNMF.

Features	Size	LSVM	QSVM	CSVM	MGSVM	CGSVM	LDA	ESD
Inception								
Pool	2048	96.0	96.1	95.8	95.7	95.6	95.6	95.5
Pool_NNMF	40	95.8	95.2	94.9	95.1	95.1	94.2	93.9
FC	8	96.1	95.9	94.7	95.7	95.8	95.3	95.0
Combined (Pool_NNMF + FC)	48	96.7	96.3	95.7	96.1	96.0	95.5	95.4
ResNet101								
Pool	2048	96.5	96.3	96.3	95.7	95.8	95.8	96.1
Pool_NNMF	40	95.6	95.0	94.5	94.6	95.1	94.3	94.0
FC	8	96.2	95.9	95.2	96.0	96.0	95.3	95.0
Combined (Pool_NNMF + FC)	48	96.7	96.4	95.5	95.9	96.4	95.8	96.0
Xception								
Pool	2048	96.2	96.2	96.2	95.7	96.0	95.5	95.8
Pool_NNMF	40	95.3	95.3	94.5	94.8	95.1	94.5	94.1
FC	8	96.4	96.0	95.2	96.0	96.1	95.9	95.5
Combined (Pool_NNMF + FC)	48	96.5	96.3	95.7	95.7	96.3	95.8	96.1

**Table 7:** Classification accuracies (%) for the HyperKvasir dataset after combining both FC and Pooling features after applying NNMF.

Features	Size	LSVM	QSVM	CSVM	MGSVM	CGSVM	LDA	ESD
Inception								
Pool	2048	96.8	96.9	96.9	96.7	96.5	96.3	96.6
Pool_NNMF	50	96.7	96.4	95.8	96.1	96.6	96.0	95.8
FC	10	97.0	96.9	96.0	96.8	96.3	96.1	95.7
Combined (Pool_NNMF + FC)	60	97.3	97.3	96.9	96.8	97.1	96.8	96.9
ResNet101								
Pool	2048	96.9	96.8	96.8	95.9	96.2	96.3	96.6
Pool_NNMF	40	96.2	95.9	95.5	94.8	95.6	94.8	94.0
FC	10	97.1	96.8	96.1	96.9	96.6	96.2	96.8
Combined (Pool_NNMF + FC)	50	97.6	97.3	96.8	96.1	96.7	96.3	96.3
Xception								
Pool	2048	97.5	97.4	97.3	97.0	97.3	97.1	97.2
Pool_NNMF	50	96.9	96.7	95.8	95.8	96.5	97.0	96.6
FC	10	97.7	97.3	96.4	97.3	97.5	97.6	97.5
Combined (Pool_NNMF + FC)	60	97.7	97.6	97.2	96.8	97.5	97.6	97.6

**Table 8:** Accuracy rates (%) obtained in different classifiers after the selection of combined deep features from all CNN models with mRMR for the Kvasir v2 dataset.

# Features	LSVM	QSVM	CSVM	MGSVM	CGSVM	LDA	ESD
10	96.1	95.9	94.7	95.8	95.8	95.5	95.1
20	97.4	97.1	96.4	97.1	97.4	97.0	96.8
30	97.6	97.3	96.9	97.4	97.5	97.0	97.0
40	97.7	97.4	97.2	97.4	97.6	97.0	96.9
50	97.7	97.5	97.3	97.4	97.6	97.0	96.9
60	97.6	97.4	97.2	97.3	97.6	97.1	97.0
70	97.8	97.5	97.2	97.4	97.5	97.0	97.0
80	97.6	97.5	97.2	97.4	97.6	97.2	97.2
90	97.8	97.7	97.3	97.5	97.7	97.2	97.2
100	97.7	97.6	97.5	97.4	97.6	97.1	97.3

**Table 9:** Accuracy rates (%) obtained in different classifiers after the selection of combined deep features from all CNN models with mRMR for the HyperKvasir dataset.

# Features	LSVM	QSVM	CSVM	MGSVM	CGSVM	LDA	ESD
10	96.4	95.9	95.0	95.9	95.9	95.2	94.9
20	98.0	97.9	97.2	97.7	97.4	97.7	97.5
30	98.2	98.1	97.8	97.8	98.0	98.3	98.1
40	98.2	98.3	97.9	97.9	98.1	98.3	98.2
50	98.3	98.1	98.0	97.9	98.0	98.2	98.1
60	98.2	98.1	97.9	97.8	98.0	98.2	98.2
70	98.3	98.2	98.1	97.8	98.0	98.1	98.1
80	98.2	98.2	98.0	97.6	97.9	98.0	98.1
90	98.2	98.2	97.9	97.7	98.0	98.0	98.0
100	98.4	98.1	98.0	97.7	98.0	98.1	98.2

**Table 10:** Performance metrics for the ML classifiers after feature selection using the mRMR approach (highest performance) for Kvasir v2 and HyperKvasir datasets.

Classifier	Sensitivity	Specificity	Precision	F1-Score	MCC
Kvasir v2					
LSVM	0.9779	0.9969	0.9781	0.9779	0.9749
QSVM	0.9766	0.9965	0.9768	0.9766	0.9732
CSVM	0.9745	0.9960	0.9747	0.9745	0.9710
MGSVM	0.9749	0.9961	0.9750	0.9749	0.9714
CGSVM	0.9765	0.9964	0.9765	0.9765	0.9729
LDA	0.9718	0.9956	0.9719	0.9718	0.9678
ESD	0.9729	0.9958	0.9729	0.9729	0.9689
HyperKvasir Dataset					
LSVM	0.983	0.998	0.984	0.983	0.981
QSVM	0.982	0.998	0.983	0.982	0.980
CSVM	0.982	0.998	0.983	0.982	0.980
MGSVM	0.980	0.998	0.981	0.980	0.978
CGSVM	0.979	0.998	0.980	0.979	0.977
LDA	0.981	0.998	0.982	0.981	0.979
ESD	0.980	0.998	0.981	0.980	0.978

**Table 11:** State-of-the-art comparison using the Kvasir-v2 and HyperKvasir datasets.

Study	Method(s)	Accuracy
[30]	5-fold cross-validation with 3 new CNN models, followed by stacking ensemble (ML classifier at the second level) and McNemar statistics test	Kvasir v2: 98.42% HyperKvasir: %98.53
[31]	Combining three pre-trained models (DenseNet201, Inception V3, ResNet50) with ensemble method (model average and weighted average)	Kvasir v2: Model Average: %92.96, Weighted Average: %95.00
[33]	Image enhancement (contrast enhancement), segmentation with deep saliency maps, MobileNet-V2-based transfer learning, hyperparameter adjustment with Bayesian optimization, attribute extraction with average pooling, attribute selection with hybrid whale optimization algorithm, classification with Extreme Learning Machine	Kvasir v2: 98.02%
[35]	EfficientNet-B0, ResNet101v2, InceptionV3, InceptionResNetV2 with Intelligent Learning Rate Controller (ILRC); transfer learning, layer freezing, fine-tuning, residual learning, regularization techniques	Kvasir v2: 98.06%
[36]	Integrates case-specific dynamic weighting with Grad-CAM, leveraging three CNNs: DenseNet201, InceptionV3, and VGG19.	Kvasir v2: 91.00%
[37]	Improved StarGAN + Perspective Transformation Module + Viewpoint Attention Module + EfficientNetB7	Kvasir v2: 95.25%
[38]	-SC-DSAN(SparseConvolutionalDenseNet201+Self-Attention) -CNN-GRU -Network-levelfusion -Featureselection:EMPA -Hyperparameteroptimization:BayesianOptimization -Classification: Shallow Wide Neural Network (SWNN)	Kvasir v2: 95.10%
[39]	EfficientNet B3 + explainable AI	Kvasir v2: 94.25%
[40]	Discrete Wavelet Transform + Deep CNN (2-stage)	Kvasir v2: 97.25%
[43]	Automatic and modular pre-processing (edge removal, contrast enhancement, filtering, color mapping, scaling, gamma correction); NASNet DL model	Kvasir v2: 97.35%
[48]	ResNet-50	HyperKvasir: 94.75%
[54]	Teacher–student Framework	HyperKvasir: 89.3%
[55]	Inception + Class Imbalance Loss	HyperKvasir: 91.55%
Proposed EndoNet	Combining two deep features from two layers of three CNNs and reducing their dimensions using NNMF and mRMR methods	Kvasir v2: 97.8% HyperKvasir: 98.4%

## Data Availability

The dataset is cited in the manuscript. The data access link is https://www.kaggle.com/datasets/plhalvorsen/kvasir-v2-a-gastrointestinal-tract-dataset (accessed on 10 June 2025).
